# Rolling out Plaque-2-seq: a single plaque sequencing approach enabling rapid, low-cost sequencing of phages directly from plaques

**DOI:** 10.1099/mgen.0.001672

**Published:** 2026-04-24

**Authors:** Slawomir Michniewski, Rebecca Glenny, Andrew Kinsella, Kane Porter, Kayne Wright, Sophie Harrison, Nzubechukwu I Ugokwe, Amani Alrashidi, Srwa.J. Rashid, Chisomaga Eke, Jack C.D. Lee, Faizal Patel, Theo Josephs, Arezoo Pedramfar, Max Coleman, Hasanain F.Y Al-Dahash, Gibson A. Sabuholo, Navodya S. Roemer, Hannah Sampson, Gerald N. Misol Jr, Branko Rihtman, David J. Scanlan, Elspeth Smith, Graham Stafford, Willem van Schaik, Richard J. Puxty, Edouard Galyov, Martha R. J. Clokie, Spyridon Megremis, Andrew Millard

**Affiliations:** 1 Becky Mayer Centre for Phage Research, University of Leicester, University Road, Leicester, UK; 2 School of Life Sciences, Gibbet Hill Campus, University of Warwick, Coventry, UK; 3 School of Clinical Dentistry, University of Sheffield, Sheffield, UK; 4 Department of Microbes, Infection and Microbiomes, School of Infection, Inflammation and Immunology, College of Medicine and Health, University of Birmingham, Birmingham, UK

**Keywords:** bacteriophages, genomes, genomics, long-read sequencing, sequencing, phages

## Abstract

Rapid, accurate and scalable sequencing of bacteriophage genomes is critical to advance phage therapy, build phage biobanks and understand phage genomic diversity. Current methods are based on sequencing and assembling complete bacteriophage genomes using short- or long-read technologies. However, current protocols require large DNA input and are cost-prohibitive, which limits their application to phage collections that typically are large and have low biomass. In order to address this, we have developed *Plaque-2-seq*, a robust and cost-effective workflow for high-throughput phage genome sequencing that will transform the speed and cost of attaining phage genomes. *Plaque-2-seq* combines low-input transposase-based library preparation, amplification, nanopore sequencing and optimized assembly steps tailored to phage genomes. We applied the method to phages isolated on seven genetically diverse bacterial hosts: *Escherichia*, *Pseudomonas*, *Synechococcus*, *Enterococcus*, *Klebsiella, Serratia* and *Enterobacter*. High-quality genome assemblies were validated using CheckV and benchmarked against previously sequenced phage isolates. Compared to standard Illumina sequencing, *Plaque-2-seq* offers up to ~5–10-fold savings in sequencing price for individual labs. Furthermore, it substantially decreases the time required to produce a phage genome, once a plaque is obtained. Offering the ability to routinely obtain hundreds of phage genome sequences a week, with minimal hands-on time. *Plaque-2-seq* enables systematic genomic characterization of phage isolates, facilitating taxonomic classification, for the development of large-scale phage biobanks.

**Impact Statement**

Here we have optimized a method for high-throughput sequencing of bacteriophage genomes from single plaques (*Plaque-2-seq*). We present a robust, high-throughput and cost-effective workflow. *Plaque-2-seq* combines low-input transposase-based library preparation, amplification, nanopore sequencing and optimized assembly steps tailored to phage genomes. We demonstrate the scalability of this approach by sequencing over 100 phages from multiple bacterial hosts. This marks a step change for the field, allowing phage genome sequencing to keep pace with phage isolation rates and transforming how rapidly we can explore and understand phage genomic diversity.

## Data Summary

Genomic data were submitted to the project accessions PRJEB94355 and PRJEB89903. Individual accessions are provided in supplementary tables. Code is available from https://github.com/amillard/plaque2seq. Figshare available here: https://doi.org/10.6084/m9.figshare.31362988 [1].

## Introduction

Bacteriophages, also known as phages, are viruses that selectively target and kill bacteria. All phages consist of a protein or proteolipid capsid enveloping the viral genetic material [2]. Although phages exhibit strong morphological similarities and conservation of structural proteins leading to a small number of known morphotypes [3], their genomic diversity is vast [4]. To date, the vast majority of isolated phages belong to the tailed group of dsDNA phages of the class *Caudoviricetes* [5]. Non-tailed dsDNA phages, such as *Corticoviridae* [6]; ssDNA phages, such as filamentous *Inoviridae* [7] or icosahedral *Microviridae* [8]; and RNA phages of the class *Leviviricetes* are also known [9], but are under-represented in genomic databases.

Unlike bacteria, where the 16S rRNA gene is commonly used as a conserved marker gene for taxonomic assignment, bacteriophages have no universal conserved genes that can be utilized for rapid genomic assessment. Several sets of signature genes, such as *polA, polB*, *g20* and *g23*, have been used where prior knowledge of the expected phage type exists [10]. However, the only way to determine if a newly isolated phage is different from previously described phages is to sequence its genome. Phage genome sequencing is commonplace, with >30,000 genomes from cultured phage isolates in public databases [4]. To date, the vast majority of these have been sequenced with Illumina sequencing methods [4]. Long-read sequencing methods, such as Oxford Nanopore Technology (ONT) and PacBio, have also been used to sequence phage genomes and metagenomes [11,13].

With general renewed interest in phages and specific interest in phage therapy, there is an increasing need for high-throughput phage genome sequencing. In the context of phage therapy, particularly during the discovery phase, sequencing large numbers of phages is essential to capture their full genetic diversity. This ensures that the most effective and therapeutically promising phages are rapidly identified, prioritized and developed for clinical application [14,15]. The greater the initial diversity of the set of phages that are characterized, the greater the chances of identifying phages that have the desired characteristics. Furthermore, excluding phages that contain genes encoding for virulence factors, antimicrobial resistance or lysogeny-associated proteins can only be done by genome sequencing [16,17]. The long turnaround times of commercial sequencing services, combined with the need to generate high-titre phage stocks to obtain sufficient DNA, mean that often preliminary phenotypic characterization is carried out before genome data are available. This approach is inefficient, as phages may later be excluded due to redundancy or the presence of undesirable genes once sequencing is complete. There is, therefore, a pressing need for rapid, scalable and cost-effective sequencing methods that enable early genomic screening of large phage collections, ensuring that only genetically distinct and therapeutically appropriate candidates progress to detailed characterization.

Obtaining sufficient quantities of high-quality phage DNA remains one of the key bottlenecks in genome sequencing. Commercial sequencing suppliers typically require hundreds of nanograms of DNA [18], which means high-titre phage stocks and at least millilitre-scale lysates [19,21]. Although this is fine for a few isolates, it is not sustainable when hundreds of phages need to be processed in parallel. Illumina-based tagmentation protocols can, in principle, use a nanogram of DNA, but access to in-house sequencing facilities where such low limits can be flexibly applied is not readily available to most researchers. The Oxford Nanopore’s MinION platform offers a portable and cost-effective alternative, but similar DNA input requirements to commercial Illumina suppliers limit the technology in its current form when using tagmentation or ligation-based library approaches, as providing sufficient quantity of DNA is a limiting factor. Both platforms also share inherent limitations in that tagmentation-based library preparation excludes ssDNA phages, and many phage genomes contain base modifications that inhibit enzymatic library preparation [22,24]. Together, these factors slow our ability to systematically sequence and compare large, diverse phage collections.

Overcoming these barriers is critical if genome sequencing is to become the *starting point* of phage characterization rather than its endpoint. A workflow that enables rapid, low-cost sequencing directly from small-scale lysates would allow early exclusion of redundant or undesirable isolates, incorporation of previously intractable phage types and a truly diversity-led discovery process. In the context of phage therapy, this shift is transformative, allowing genetic insight to guide isolation and formulation from the outset, rather than retrospectively. Our goal, therefore, was to develop a sequencing approach that is scalable, accessible and cost-efficient, with minimal hands-on time and capital investment, which will transform phage genomics from being a specialist bottleneck into a routine, enabling a step change in therapeutic phage development.

## Methods

### Bacterial isolates

Bacterial strains used in this study are detailed in Table 1.

**Table 1.** Bacterial hosts used in this study

Bacterial host	Accession no.
*Escherichia coli* MG1655	U00096
*Pseudomonas aeruginosa* G03624	n/a
*Pseudomonas aeruginosa* LEI0034	n/a
*Synechococcus* sp. WH7803	NC_009481
*Enterococcus faecium* E1162	GCA_000172675
*Enterobacter asburiae*	n/a
*Klebsiella variicola*	n/a
*Serratia marcescens*	n/a
*Enterobacter cloacae*	n/a

### Phage isolation against *Pseudomonas aeruginosa and Escherichia coli MG1655*

Plaque assays were performed using a standard protocol [18] with *P. aeruginosa* as the host, using water from a pig trough. The resulting plaques were picked using a 1-ml pipette tip into separate 1.5-ml Eppendorf tubes containing 100 µl SM buffer (200 mM NaCl_2_, 10 mM MgSO_4_, 50 mM Tris-HCl and pH 7.5), which was then vortexed and stored at 4 °C for further phage purification and/or propagation. A subsequent spot test using 10 µl of the previous sample was performed to confirm whether the plaques contained phages by the formation of a zone of clearing. Finally, a soft agar plug was collected from the middle of the resultant clearing zone using a 1-ml pipette filter-tip, transferred to a fresh 1.5-ml Eppendorf tube containing 100 µl SM buffer, vortexed and left for 1 h for phages to disperse from the agar. Subsequently, the sample was centrifuged at 13,000 g for 5 min, and 10 µl of the supernatant was transferred to a well in a 96-well plate. Phages against *E. coli* MG1655 were collected similarly, using water from Barston Sewage Works as the sample source.

### DNase I treatment prior to whole-genome amplification

Each well within a 96-well plate was supplemented with 0.01 U of DNase I (Thermo Scientific) and incubated at 37 °C for 60 min, followed by 95 °C for 10 min, to ensure complete inactivation of the DNase I and simultaneous release of encapsidated phage DNA. Phage DNA samples were stored at −20 °C until further use.

### Whole-genome amplification and debranching of the products

Whole-genome amplification of phage DNA samples was performed in a separate 96-well plate using the EquiPhi29™ DNA Amplification Kit (Thermo Scientific) and following the manufacturer’s guidelines for standard 20 µl reactions, with reaction volumes reduced proportionally to half, one-third or a quarter of the standard reaction. Debranching of amplified DNA was performed by supplementing each well in a 96-well plate with 10 U of S1 nuclease (Thermo Scientific) containing buffer (1× final concentration) and incubating at 25 °C for 30 min, followed by 95 °C for 10 min, to ensure complete inactivation of the S1 nuclease. DNA concentration was quantified using a Qubit Broad Range Kit (Thermo Fisher). Amplified phage DNA samples were stored at −20 °C until further use.

### Sequencing and genome assembly

ONT sequencing was carried out using the Rapid sequencing DNA V14 - barcoding kit (SQK-RBK114.24 or SQK-RBK114.96) on a MinION Mk1c/Mk1B or GridION, with R10.4.1 flow cells. Basecalling was carried out using the High-accuracy v4.3.0–400 bps model. Reads were trimmed for barcodes and adapters after sequencing, either within MinKnow or using Dorado [19]. Long-read assemblies were carried out with flye v2.8.1-b1676 using the following settings: ‘–interations 2, –nano_corr’. Followed by two rounds of polishing with medaka using the relevant model [19]. Genome assembly was carried out on the ALICE High Performance Computing facility at the University of Leicester, with 48 cores allocated for assembly.

### Genomic analysis

#### *In silico* dataset

Long reads were generated against a set of 196 phage genomes (Table S1, available in the online Supplementary Material), which were representative of all families of DNA viruses known to infect bacteria in the ICTV VMR_v40.1 . Long reads were generated using PBSIM3 [20] with the following settings ‘--strategy’, ‘wgs’, ‘--method’, ‘--errhmm’, model_path, ‘--depth’, ‘70’, ‘--length-mean’, ‘2500’, ‘--length-max’, ‘15000’, ‘--accuracy-mean’, ‘0.99’, ‘--accuracy-min’, ‘0.80’, ‘--length-sd’, ‘5000’. Short reads were then created from these long reads using a Biopython script. Prior to assembly, reads were normalized with bbnorm.sh target=150 [21]. Genomes were assembled with SPAdes v3.13.1 with ‘-s’, for single-read libraries [22]. Assembled genomes were compared against the reference genome with dnadiff [23] to identify SNPs and insertions/deletions. Completeness of genomes was determined by the formula: Assembly Length/Reference Sequence Length × 100

### Assembly pipeline

Reads were basecalled using Dorado v.0.9.0 with ‘basecaller hac --kit-name’ and fastq reads as output. Any chimeric reads were identified and split with SACRA v.2.0 [24]. The resulting split reads were initially assembled with Flye v2.9.5 using ‘nano_corrected’. Assembled contigs were polished twice with Medaka v2.0.0 using ‘medaka_consensus’, using the relevant model. Phage genomes within each contig file were identified with CheckV v1.0.3 ‘end_to_end’ to rapidly identify complete phage genomes [25]. All ‘Complete’, ‘High-Quality’ and ‘Medium’ genomes were extracted, and taxonomy was assigned to complete and high-quality genomes using *taxMyPhage* [26].

### Short-read assembly

Long reads were converted to short reads of length 300 bp with a Python script. Assembly was then carried out as previously described [27], with minor modifications. Briefly, reads were normalized with bbnorm ‘--target=150’, prior to assembly with SPAdes v3.13.1 ‘-s, -- only-assembler’, allowing assembly with single-end reads. All ‘Complete’, ‘High-Quality’ and ‘Medium’ genomes were extracted, and taxonomy was assigned to complete and high-quality genomes using ta*xMyPhage*t [26]. Scripts used for genome assembly are available on GitHub (https://github.com/amillard/plaque2seq).

### Quality control of sequencing data

The proportion of reads assumed to be host-derived was determined by mapping reads against the host genome with ‘minimap2 -ax map-ont -t {threads} {ref_fa} {input_fq} | samtools view -Sb - | samtools sort -o {output_bam}’ . The number of mapped reads was determined with pysam [28] and calculated as a percentage of total reads. *Pseudomonas* prophages were predicted with geNomad ‘end-to-end’ [29], and these regions were manually excised from the host genome to create a prophage-free reference, which was then used to calculate the percentage of mapped host reads.

### Genome annotation, taxonomic classification and comparative genomics

Genomes were annotated with Pharokka v1.7.5 [17] using PHROGS HMMs [30]. All genomes were submitted to the European Nucleotide Archive [see supplementary tables for the accession numbers (Table S2)]. Bacteriophages were classified into existing genera and species utilizing *taxMyPhage* v0.3.3, using default settings [26]. Bacteriophages were compared to each other using the *taxMyPhage* ‘similarity’ option, with genomes identified as 100% ANI, classified as identical.

### ‘Warwick’ and ‘Sheffield’, phage isolation, genome sequencing and assembly

To validate the approach, the protocol was carried out by researchers at the University of Warwick and Sheffield

### Phage isolation against *Synechococcus* sp. WH7803

Cyanophages were isolated from seawater samples against *Synechococcus* sp. WH7803 according to standard protocols [31]. Initially, 200 µl of seawater sample from the Atlantic Meridional Transect 24 (AMT24) research cruise was inoculated into a 1.8-ml *Synechococcus* sp. WH7803 culture and incubated for 2 weeks at 22 °C at a continuous light intensity of 10 µmol photons m^2^ s^−1^. The infected cultures were then centrifuged at 4,000 ***g*** for 10 min and filtered through 0.2 µM pore size filters to obtain the phage fraction. Subsequently, 20 µl aliquots of the phage fraction were spotted onto *Synechococcus* sp. WH7803 lawns to screen for phage-producing samples. Samples that produced a zone of clearing were resuspended in 1 ml ASW medium [31]. To obtain clonal phages using plaque assays, 10 µl of the resuspension was added to 100 µl of 10× concentrated *Synechococcus* sp. WH7803 and was incubated for 1 h at 22 °C with illumination. The infection was then mixed with 3 ml cooled molten ASW medium agar (0.2% w/v) and incubated as above. After incubation, single plaques were picked and resuspended in 1 ml ASW medium. This process was repeated twice more to obtain clonal cyanophages. Clonal cyanophages were subsequently used for sequencing as described above, with the omission of the S1 nuclease step. Phage genomes were sequenced on a MinION Mk1C. Reads were base called using Dorado v 0.9.0 with ‘basecaller hac --kit-name’, with fastq reads as output. Genomes were assembled from short reads as described above, and the resulting genomes were annotated with Pharokka using the PHROGs database, as previously described [27,30, 32]

### Phage isolation against *Enterobacter, Enterococcus, Klebsiella* and *Serratia*

Phages were isolated from wastewater samples collected from Blackburn Meadows, Sheffield and animal faeces donated by Yorkshire Wildlife Park, Doncaster. One hundred fifty microlitres of filtered (0.45 µm pore size) environmental samples were mixed with 0.5 ml of bacterial culture (OD 0.5–1) and incubated for 10 min at room temperature. After incubation, 5 ml of BHI soft agar (0.4% w/v) (Avantor, USA) was added to the mixture, inverted to mix and then immediately poured on top of a solid BHI agar plate and incubated overnight at 37 °C. Plaques were then picked and resuspended in 50 µl of PBS and stored at 4 °C until re-infection. To ensure the purity of the possible phage, three successive rounds of plaque picking and plaque assays were carried out. For testing the *Plaque-2-sequence,* a single plaque was picked and resuspended in 100 µl of PBS, vortexed and left for 1 h to resuspend. The plaque resuspensions were then used for sequencing as described above, with the omission of the S1 nuclease step.

Reads were basecalled with Dorado v 0.9.0 with ‘basecaller sup --kit-name’, with fastq reads as output. The phages *Klebsiella* phage Kv_Kong and *Enterococcus* phage Efm_George were sequenced by both *Plaque-2-seq* and standard extraction of DNA from high-titre lysate (un-amplified). For reads generated from unamplified DNA, assemblies were carried out on the Galaxy web platform (https://usegalaxy.eu). Fastq reads were assembled using Raven v1.8.3 with default parameters [33], and assembled contigs were polished with the Medaka Consensus Pipeline v1.7.2 using the model ‘r1041_e82_400bps_sup_g615’ (https://github.com/nanoporetech/medaka). The resulting contigs were analysed with CheckV v1.0.3 to identify complete phage genomes [25]. Genomes were annotated with Pharokka v1.3.2 [17]. Phage SM58.13_P2 was also assembled using the approach described above. All other genomes generated from amplified DNA were assembled by the previously described approach of creating short reads from long reads, with the exception of *Serratia* phage SM58.13_P2, which did not produce an assembled genome from short reads. Host contamination was calculated by mapping reads to the complete phage genome with Minimap2 v2.28 [34]. Host reads were calculated as those that did not map to the phage genome.

## Results

To remove the labour-intensive step of multiple rounds of plaque purification and production of high-titre stocks for DNA extraction, we tested the use of whole-genome amplification using multiple displacement amplification (MDA) of phage DNA to provide sufficient DNA for sequencing from a single plaque. As the aim was to develop a process which was as cost-effective as possible, we tested 0.5×, 0.3× and 0.25× volumes relative to the 20 µl recommended volume by the manufacturer of EquiPhi29 MDA kits, as well as testing 1, 2 and 4 h incubation times with a single plaque as input material. There was no significant difference in the yield of DNA obtained under the nine conditions tested, with all producing sufficient DNA for sequencing (Fig. S1). Thus, 0.25 volumes of the recommended amount were used in further experiments (Fig. S1).

Having established that DNA could be amplified from a single plaque, we next determined if different treatments were required to successfully assemble phage genomes from the obtained DNA. As the process was based solely on touching a plaque with a sterile tip, there is a high probability of carry-over of host DNA. Furthermore, the process of MDA amplification is known to produce chimeric branching into DNA [24]. Thus, we designed an experiment whereby we could assess if these factors might impact our results. In short, 48 single plaques were obtained, using a single water sample from a pig trough, against a clinical isolate of *P. aeruginosa*. All plaques were resuspended in 100 µl of SM buffer and treated using four different protocols, prior to sequencing (Table 2).

**Table 2.** Treatments applied to 48 plaques obtained against *P. aeruginosa*

	DNase I treatment	Amplification	S1 nuclease	Gb of data
Treatment 1	N	Y	N	9.65 Gb
Treatment 2	Y	Y	N	2.84 Gb
Treatment 3	N	Y	Y	7.53 Gb
Treatment 4	Y	Y	Y	9.14 Gb

The chosen method of sequencing was ONT, due to the minimal capital costs associated with the equipment. Of the expected 192 samples, DNA was obtained from 174 samples, with failed samples due to edge effects associated with incomplete sealing of 96-well PCR plates. For each treatment group, 1 sequencing library was produced, with up to 48 barcodes per flow cell. Each library was sequenced on a different R10.4.1 flow cell.

### Sequencing results

**Fig. 1.**
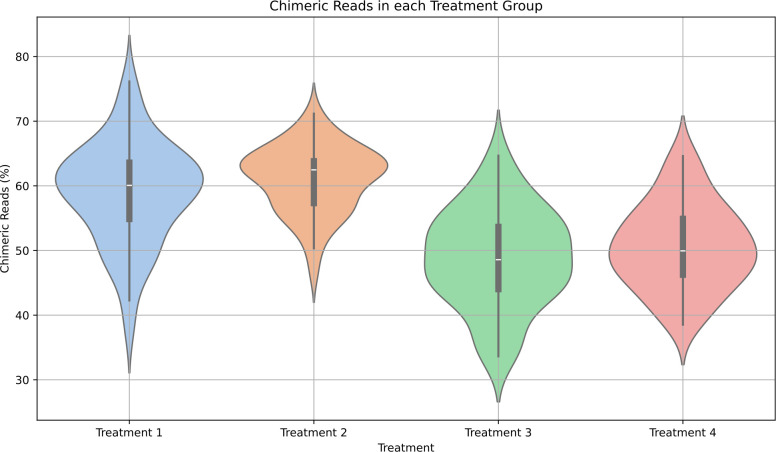
Detection of chimeric reads in four test conditions. The percentage of chimeric reads was determined from the output of SACRA [24]. Treatment 1, MDA only nuclease; treatment 2, MDA and DNAse; treatment 3, MDA and S1 nuclease; treatment 4, MDA, S1 nuclease and DNase I.

The total amount of sequencing data produced per treatment (174 plaques) varied from 2.84 to 9.65 Gb of data (Table 2). The largest yield of sequencing data was obtained from treatment group 1 (9.65 Gb), closely followed by groups 4 (9.14 Gb) and 3 (7.53 Gb), with data output from treatment 2 (2.84 Gb) being the lowest. As MDA is known to introduce chimeric reads [24], we calculated the percentage of reads that contained putative chimaeras for each treatment. In treatments 1 and 2, during which no S1 nuclease was applied, the median percentage of chimeric reads was 60.2 and 62.7 (Fig. 1). There was a significant decrease (Mann–Whitney *U* test, with Benjamini–Hochberg correction, *P*<0.05) in the percentage of chimeric reads in treatments 3 and 4 with 48.5 and 49.9, respectively. Thus, the inclusion of S1 nuclease in our protocol reduced the number of chimeric reads, but did not eliminate all chimeric reads.

To determine the effect of DNase I treatment, reads were mapped against the host *P. aeruginosa* reference genome sequence to calculate the percentage of contaminating host reads. However, >80 samples were found where >90% of reads mapped to the host genome, suggesting no phages were present, high levels of induced prophages or a combination of both. To overcome this, prophage regions were predicted with geNomad in the host genome, and these regions were excluded from inclusion in the calculation of reads mapped to the host genome. However, a high percentage of reads was still observed across all samples, with no significant differences in the proportion of reads mapped to the host genomes across the four treatment groups (Figure S2). The optimal conditions for DNA amplification and sequencing were utilizing 0.25× volumes of MDA reagents with an S1 nuclease step. As DNase I was not detrimental and had minimal cost, we included this in further work.

### Bioinformatics optimization

**Fig. 2.**
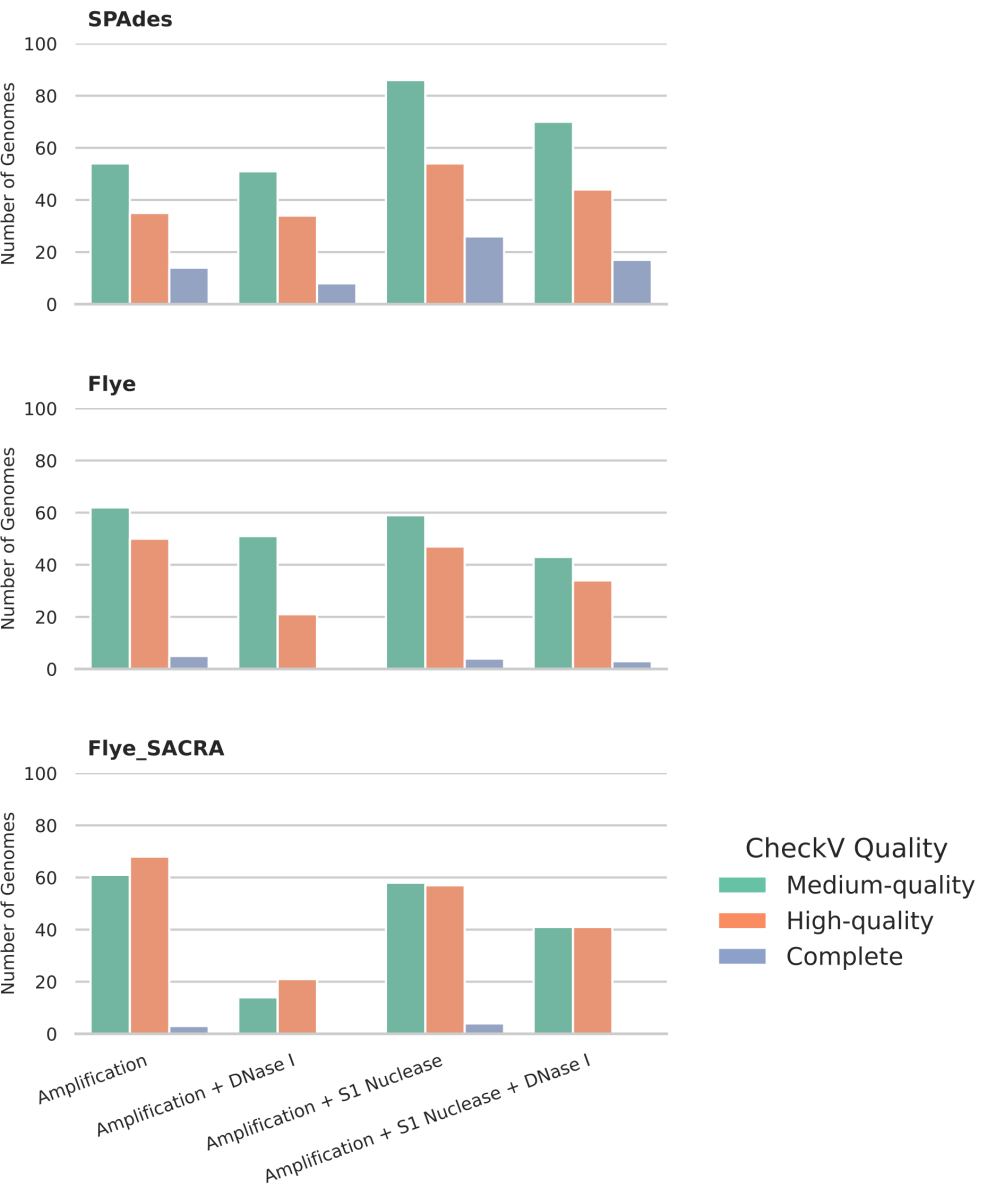
Comparison of assembly approaches. Three different assembly approaches were compared: SPAdes, assembly of short reads; Flye, assembly of long reads; Flye+SACRA, assembly of long reads with detection and splitting of chimeric reads with SACRA [24]. The number of phage genomes in each treatment group was determined using CheckV [25]. Treatment 1, MDA, S1 nuclease only; treatment 2, MDA and DNase I only; treatment 3, MDA and S1 nuclease only; treatment 4, MDA, S1 nuclease and DNase I.

We next sought to automate the assembly of phage genomes. We utilized Flye for the assembly of long reads, polishing with medaka and CheckV for the automated identification of phage genomes within each sample (Fig. 2). We compared this process with an additional step of utilizing SACRA for the identification and splitting of chimeric reads [24], prior to assembly. Although 48 plaques were selected, due to the aforementioned failed DNA amplification or failed sequencing, only 39/48 samples were present in all treatment groups. Only samples present in all four treatment groups were included in the comparative genome analysis. The number of complete, high-quality and medium-quality genomes was assessed with CheckV.

Across all conditions, the use of Flye+SACRA generally increased the number of contigs that could be identified as phage within the assemblies (Fig. 2). A comparison of the ‘Flye alone’ and Flye+SACRA assembly approaches, on samples where no DNase I or S1 nuclease step was applied (treatment 1), showed that a greater number of predicted complete and high-quality genomes were observed utilizing SACRA to split chimeric reads (Fig. 2). A similar pattern was observed even when both S1 nuclease and DNase I were utilized (treatment 4), with the combined number of high quality and complete genomes increasing from 37 when using Flye alone to 41, when utilizing Flye+SACRA (Fig. 2). While SACRA was useful in the identification of chimeric reads and resultant assemblies, it had an additional cost of significantly increased computational time. The assembly of 48 genomes when utilizing SACRA was ~36 h, compared to <12 h without.

To reduce the time for assembly, we hypothesized that turning long reads into short reads and using a short-read assembler would decrease the assembly time. We first tested if creating short 300 bp reads from long reads and then assembling with the short-read assembler SPAdes would allow recovery of complete phage genomes. To test the applicability of this approach, we first created a simulated set of long reads from a representative set of bacteriophage genomes (Table S1) that were then made into short reads. The percentage completeness (compared to the original reference) for this *in silico* dataset ranged from 83.39 to 100.1%, with a median of 99.79% (Fig. S3), thus demonstrating the approach works for simulated reads.

When applied to the data generated in this study, the largest number of complete and high-quality genomes in all conditions was observed when utilizing SPAdes (Fig. 2). Complete and high-quality predictions have been combined for comparison, as CheckV utilizes terminal repeats to classify complete genomes from high quality. However, SPAdes introduces repeated kmers at the ends of circularly permuted contigs, which are classified as complete, whereas Flye does not introduce such kmers to identify circular contigs. Any circularly permuted genomes will be called complete if assembled by SPAdes, but not by Flye, even if both are complete. Thus, overall, we recommend creating short reads from long reads and using SPAdes for assembly, as it maximizes the number of genomes, with the lowest computational time.

The automated assembly and phage identification approach identified many more than the 39 genomes, which would be expected if each plaque represented a single phage (Fig. 2), with 80 genomes in treatment group 3 using SPAdes assembly. The reason for this is the assembly (and partial assembly) of prophages from the host organism, which were assembled at the same time as any isolated phages. Prophages were identified by comparison against the host genome. Of the original 48 plaques across all 4 treatments, it was possible to determine 19 solely contained prophages, suggesting they were caused by spontaneous induction of host prophages. Four plaques contained 2 phages, other than prophages, and 21 plaques were caused by other phages (although still contained prophage DNA). Thus, this approach allowed the rapid identification of 29 new phage isolates.

### Scaling up to 96 barcodes

**Fig. 3.**
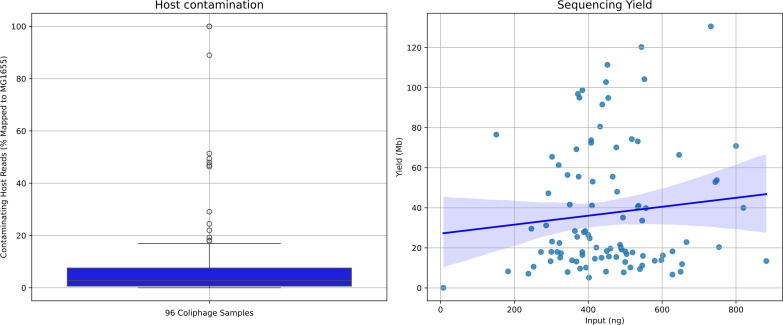
Output of sequencing data for 96 coliphage samples. (**a**) Boxplot of host contamination for 96 coliphage samples. Host contamination was determined by mapping of reads to *E. coli* MG1655. (**b**) Sequencing yield (Mb) plotted against library input (ng) for 96 coliphage samples.

Having established that short reads, derived from long reads, can be utilized for single plaque sequencing, we further tested the approach with *E. coli* MG1655 as a host, increasing the number of plaques tested to 96 from a single water sample, completing the entire process within a week (Fig. S4). We utilized both an S1 nuclease step, as this reduced chimeric reads and DNAse I treatment in library preparations (Fig. S5). Of these 96 samples, 95 produced sufficient DNA for further sequencing. The approach of MDA was remarkably consistent with a median yield of 443 ng µl^−1^ (±146 ng). As *E. coli* MG1655 has only cryptic prophages [35], the levels of host DNA per library were readily quantifiable by read mapping to the host chromosome. The median host DNA contamination was 2.75% (±19%) per sample. Sequencing yield per sample varied across samples, with no correlation of input amount and output yield (Fig. 3). Despite this variability, it was sufficient to provide the depth of sequencing required for a single phage genome. Assuming a mean phage genome size of 75 kb, the median predicted depth of sequencing would be 302× per phage genome, which far exceeds the ~30× coverage needed for genome assembly [36].

The resulting assembly of 87 samples into 91 genomes confirms sufficient coverage was obtained. For samples which did not result in the production of a phage genome (*n*=9), one of these was a result of insufficient DNA amplification, and two were from amplification of solely bacterial DNA. The failure of the other six samples was not clearly identifiable. Four samples were found to have two phages present. The rapid sequencing of plaques from a single source material also rapidly identified the diversity of phages present within a single sample. Of the 91 coliphage genomes assembled, 42 of the 91 were unique when compared against each other (Fig. S6). From this single source of wastewater used for isolation, phages classified into two families (*Drexlerviridae and Straboviridae*) and seven genera were isolated (*Dhillonvirus*, *Felixounavirus*, *Hanrivervirus*, *Krishvirus*, *Tequatrovirus*, *Vequnitavirus* and *Warwickvirus*) (Table S2).

### Genome fidelity

In order to test the fidelity of the process, we resequenced the *Salmonella* phage RA112 using nanopore reads and compared this to the original Illumina assembly, resulting in an identical genome sequence. Furthermore, we sequenced three plaques from the same coliphage (CCE_0049) and obtained identical sequences (Table S2). Repeating a similar process using *Pseudomonas* phage GAS_0134 produced identical results, and the ssDNA *Pseudomonas* phage GAS_0134B also produced identical genomes, again confirming the reproducibility and accuracy of the approach.

### Independent validation

Having established the protocol at the Becky Mayer Centre for Phage Research at the University of Leicester, the approach was independently carried out at the University of Warwick and the University of Sheffield. Using the approach, a further 24 plaques obtained on *Synechococcus* sp. WH7803 were sequenced at the University of Warwick. A total of 21 genomes were obtained, ranging in length from ~171 to ~226 kb. The failure of three genomes likely resulted from insufficient data, with three samples yielding <5 MB of sequence data. Why the remaining two genomes did not assemble was unclear. The mean level of contaminating host DNA reads was estimated to be 0.825%±2.6%, by mapping to the host genome (Table S3).

A further seven phages were isolated and sequenced at the University of Sheffield, isolated on *Enterococcus faecium*, *Enterobacter asburiae*, *Klebsiella variicola*, *Serratia marcescens* and *Enterobacter cloacae*, confirming the approach can be used on different bacterial species to sequence phage genomes that range in size from ~150 to ~275 kb. Phages Kv_Kong and Efm_George were sequenced using both amplified and unamplified DNA, and comparison of the resulting genomes using DNAdif [37] confirmed no differences between the two approaches. Thus, demonstrating that the approach of *Plaque-2-seq* works on plaques from a range of bacterial species and produces genomes of the same quality as other approaches. The level of host contamination was 7.19%±8.42% across the samples sequenced (Table S4).

## Discussion

We have developed an approach to rapidly sequence bacteriophage genomes that can be scaled to sequence thousands of bacteriophage genomes at greatly reduced cost. By optimizing each step, we have developed a process which allows phage genomes to be sequenced for ~£7 per genome, when applied to batches of 96 plaques (costs are based on DNA amplification and sequencing costs) (Table S5). The process can also be applied to a smaller number of genomes in batches of 24 or 48 at an increased cost of still <~£25 or individual phages with increasing cost. The approach does not require substantial capital costs, with the entry-level MinION Mk1D available for <£3,000 (May 2025). We have substantially reduced the time taken to obtain complete phage genomes, streamlining the process such that 96 plaques can be ‘picked’ on Monday with complete phage genomes obtained by Friday/Saturday by a single user (Fig. S5).

The cost savings generated by our approach could be further improved by washing the flow cell between library runs. The theoretical output of a minION flow cell is 50 Gb of data. However, the actual obtained output per flow cell is dependent on a number of factors [38]. Within this study, we achieved ~10 Gb of data from a flow cell on multiple occasions. Only 30× coverage is required for the assembly of phage genomes [36]. Assuming a genome size of 75 kb, with a 60× coverage, requires 864 Mb of data for 192 phage genomes (with no host contamination). Thus, halting a run once ~60× coverage has been achieved for 96 samples, and then washing and loading a new library with a further 96 samples offers further potential for cost savings. Sequencing 192 samples on a flow cell could reduce the cost to <£5 a genome using our approach. Further savings could be achieved by running batches of 96 barcodes on a flow cell (Table S5).

By sequencing from single plaques, we greatly reduced the time taken to obtain phage genomes, once a plaque is identified. Previously, single plaque sequencing has been optimized for Illumina sequencing by maximizing the recovery of DNA from a single plaque and minimizing the input into sequencing library preparation [39]. The previous approach does not scale well to the sequencing of hundreds or thousands of phage genomes, due to the filtration step and low input into Illumina sequencing libraries. The input for Illumina Nextera XT can be reduced to <1 ng DNA when running an Illumina machine within an individual laboratory. However, the capital costs of such machines are high, and commercial suppliers do not routinely accept such a low concentration of DNA due to their quality control processes. Recently, nanopore sequencing has been used to sequence single phage genomes from as little as 0.4 ng DNA [40]. However, while feasible, the approach is not practical for large-scale genome sequencing due to the limited number of reads that were obtained within a 72 h run on a minION. While sufficient to sequence a single genome, the output from such low input does not allow rapid sequencing of hundreds of genomes [40].

To overcome the limited DNA obtained from a plaque, MDA was utilized. The use of MDA within our approach offers both advantages and disadvantages to genome sequencing. The primary advantage is the rapid increase in DNA that allows high-throughput approaches to be adopted. Secondary to this, it enables the sequencing of ssDNA phages, such as filamentous phages belonging to the family *Inoviridae*. MDA results in the dsDNA product from the ssDNA template, which can then be used to construct a sequencing library using a common, dsDNA-specific tagmentation approach. Finally, it also removes DNA modifications from bacteriophage genomes, which are known to be extensively hypermodified and recalcitrant to standard sequencing approaches [41,43]. The quantification of DNA after MDA allows the putative identification of phages that may have modified DNA that the EquiPhi enzyme cannot process and can be investigated further. The failure to produce amplified DNA can be from the lack of DNA input or modified DNA preventing amplification. The lack of output DNA may result from isolating phages with RNA genomes and thus having no DNA to amplify or phages with DNA modifications, also preventing amplification.

The drawback of MDA is the removal of these biologically interesting DNA modifications, as they are replaced with standard nucleotides during amplification, so any modifications can no longer be detected or quantified. Additionally, amplification also removes the ability to precisely define the termini of phage genomes with approaches such as PhageTerm [44]. It should be noted that the majority of phage genomes sequenced to date have utilized Illumina sequencing with Nextera XT [4], which also prevents recovery of genomic termini [44].

The use of MDA is known to introduce chimaeras into reads [24]. The use of S1 nuclease significantly reduced the number of chimeric reads, but the percentage of chimeric reads remained high (48.5 and 49.9 in S1 nuclease-treated samples). Thus, identification and splitting of these reads were necessary with SACRA [24]. It became apparent that the limiting step in obtaining a complete genome was the time for the identification of chimeric reads with SACRA. SACRA functions by the identification of partially aligned reads compared to continuously aligned reads and the splitting of reads at a chimeric junction [24]. Although utilizing SACRA provided an improvement in the number of assembled genomes, it proved to be a time-intensive step. To speed up the process of genome assembly, we took the approach of naively splitting reads into 300 bp fragments and treating them as short reads. This approach reliably increased the number of phage genomes, which is consistent with previous studies that showed that the majority of phage genomes can be assembled from short reads [36].

We incorporated a DNase I step to test the effectiveness of reducing bacterial DNA. With the *Pseudomonas* phage set, there was no significant difference in the levels of host DNA when DNase I was used, and it was high across all samples. The presence of prophages, which may be induced, would increase the level of host contamination. To account for this, we identified prophages with geNomad and manually removed them from the host genome and calculated the percentage of mapped reads to infer host DNA contamination. The predicted percentage of mapped reads remained very high. There are likely numerous reasons for this. These include (1) not all prophages will be predicted and any remaining that are induced will be counted as host DNA using a mapping approach; (2) mapping allows sequence divergence, thus a closely related temperate phage that was isolated will have reads that map to unidentified prophages in the host chromosome. (3) Phages are capable of both generalized and specialized transduction, which will package host DNA [45,46]. We were unable to completely rule out that the different frequencies of transduction were not contributing to differences in the levels of host DNA. Despite the presence of host DNA within samples, it was still possible to assemble phages from samples, due to the relatively large amounts of data obtained. When DNase I was used with *E. coli* MG1655, which does not contain an inducible prophage and the levels of host DNA are easily quantifiable, the percentage of DNA in samples was low (<3%) and did not hinder genome assembly. The levels of host DNA contamination in the cyanophage samples and the range of hosts from the ‘Sheffield phages’ were also very low compared to the *Pseudomonas* samples. We were unable to determine if DNase I was the reason for this. However, its addition does not prevent phage genome assembly. The heating of the sample to denature the DNase I, which also releases viral DNA from capsids at the same time, does not seem to be problematic for low-input amplifications, as seen by the successful genome sequencing of phages.

In the future, the method could be further strengthened when isolating large numbers of phages on a single host by incorporating host genomic characterization prior to phage isolation. Sequencing the host genome in advance would allow *in silico* prediction of prophages. In parallel, sequencing the host culture supernatant – treated analogously to a plaque sample – would enable detection of spontaneously released prophages and help identify inducible elements that may not have been predicted bioinformatically from the genome sequence alone. Such an approach would, therefore, capture both computationally predicted and actively released prophages.

The development of a high-throughput, cost-effective approach underpins future progress to make phage therapy a viable alternative or complement to antibiotics. Recent studies have demonstrated the predictive power of using phage genomes and host genomes to select phages that will infect [47,48]. With the increasing size of datasets, the accuracy of such predictions will also increase. In selecting phages for therapy, it is desirable to know very early on in the selection of phages if they contain genes that encode for antibiotic resistance genes or virulence factors [49], to prevent detailed characterization of such phages. There have been several recent advances in the ability to rapidly isolate phages for therapeutic use [50,51]. The approach described here now allows genome sequencing to keep pace with that of isolation at a greatly reduced cost.

## Conclusions

We have developed a cheap and rapid method that will facilitate the sequencing of hundreds of phages at a greatly reduced cost. In demonstrating the approach, we showed that it can be used to sequence phage genomes, which vary in size from ~5 to 279 kb, can be dsDNA or ssDNA and work on plaques obtained from both Gram-positive and Gram-negative bacterial hosts. The use of amplified DNA removes the ability to identify modified bases and the termini of genomes. However, within the context of phage therapy, it will enable the rapid development of robust phage biobanks, built upon genomic data. The reduced cost per genome and low capital costs allow phage banks to be developed *in situ* in low and middle-income countries, where the need for phage biobanks is often greatest. The sequencing of single plaques on *E. coli* also identified only 4 of 96 plaques that contained multiple phages, demonstrating that it is not necessary to utilize three rounds of plaque purification to obtain purified phages for further characterization. Within a broader context, it will allow for a widespread expansion in both the number and diversity of phage genomes, which are greatly under-represented compared to their bacterial hosts.

## Supplementary material

10.1099/mgen.0.001672 Uncited Fig. S1.

10.1099/mgen.0.001672 Uncited Table S1.

## Abbreviations

MDA: multiple displacement amplification
ONT: Oxford Nanopore Technology
