# Supplementary material for: Rolling out Plaque-2-seq: a single plaque sequencing approach enabling rapid, low-cost sequencing of phages directly from plaques
Source: Microb Genom. 2026 Apr 24;12(4):001672. doi: 10.1099/mgen.0.001672 (PMC13108922; doi:10.1099/mgen.0.001672)
Supplement: Uncited Fig. S1. [file mgen-12-01672-s001.pdf]

Supplementary Figures

Figure S1 . Multiple displacement amplification of DNA from plaques. Twenty seven plaques were resuspended in SM buffer and MDA applied. The following conditions were applied, amplification for 1 , 2 and 4 hours and amplification using 0.5x , 0.3x and 0.25x the standard 20 ul volumes. To give a total of 9 conditions, with 3 replicates per condition. No significant difference was found for DNA yield, between conditions (one way ANOVA).

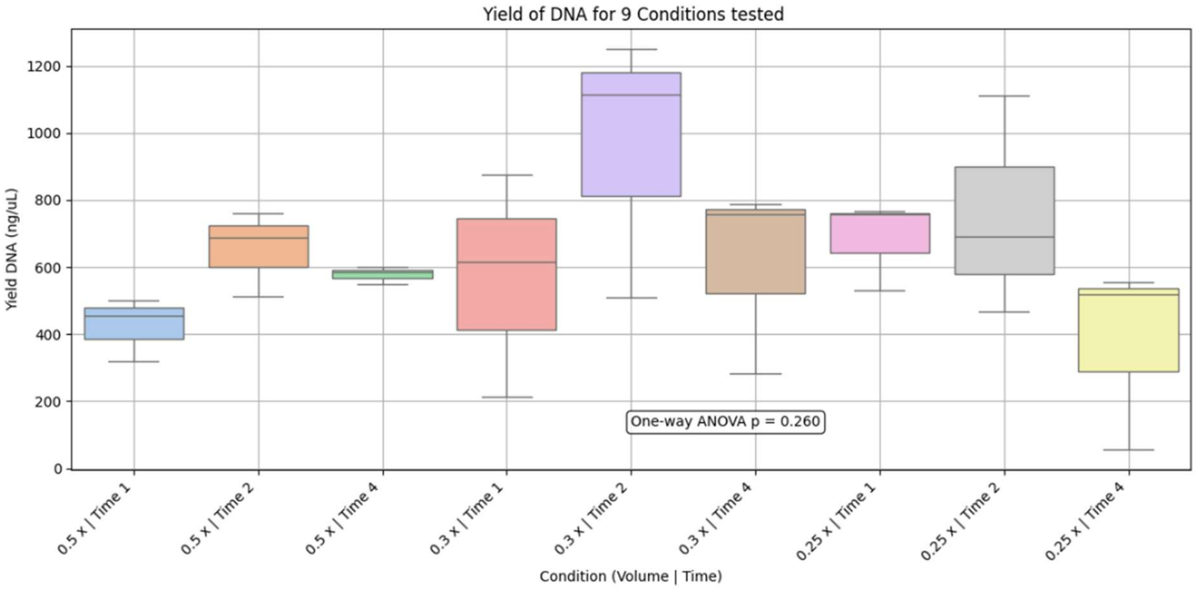

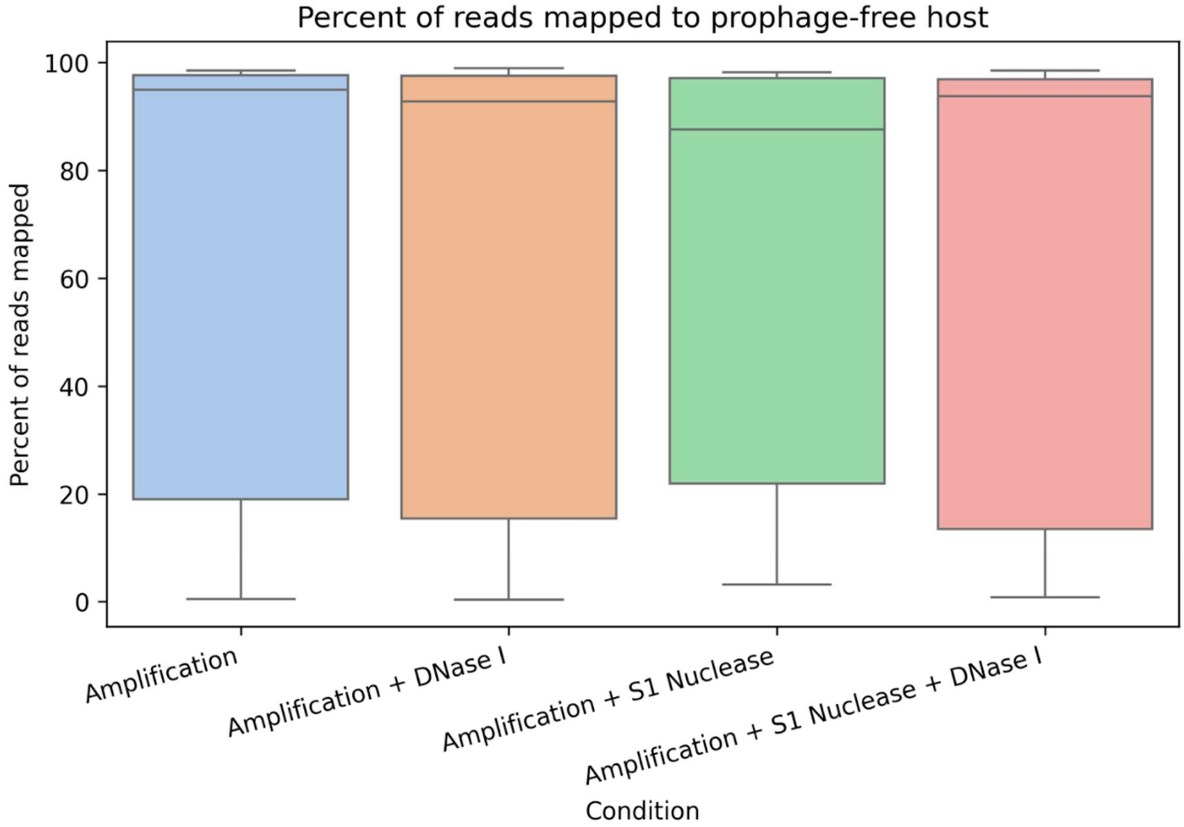

Figure S2. Detection of host DNA in four treatment groups. The percentage of host reads was determined by mapping of reads against the *Pseudomonas* host, which had predicted prophages removed. Data plotted has been filtered to only include samples where sequence was obtained in all four treatments, < 100% of reads mapped to the host (to excluded samples with no phage) and >10000 reads (removal of samples with low sequencing output). Treatment 1:MDA only S1 Nuclease; Treatment 2: MDA and DNase I; Treatment 3: MDA and S1 Nuclease; Treatment 4: MDA, S1 Nuclease and DNase I.

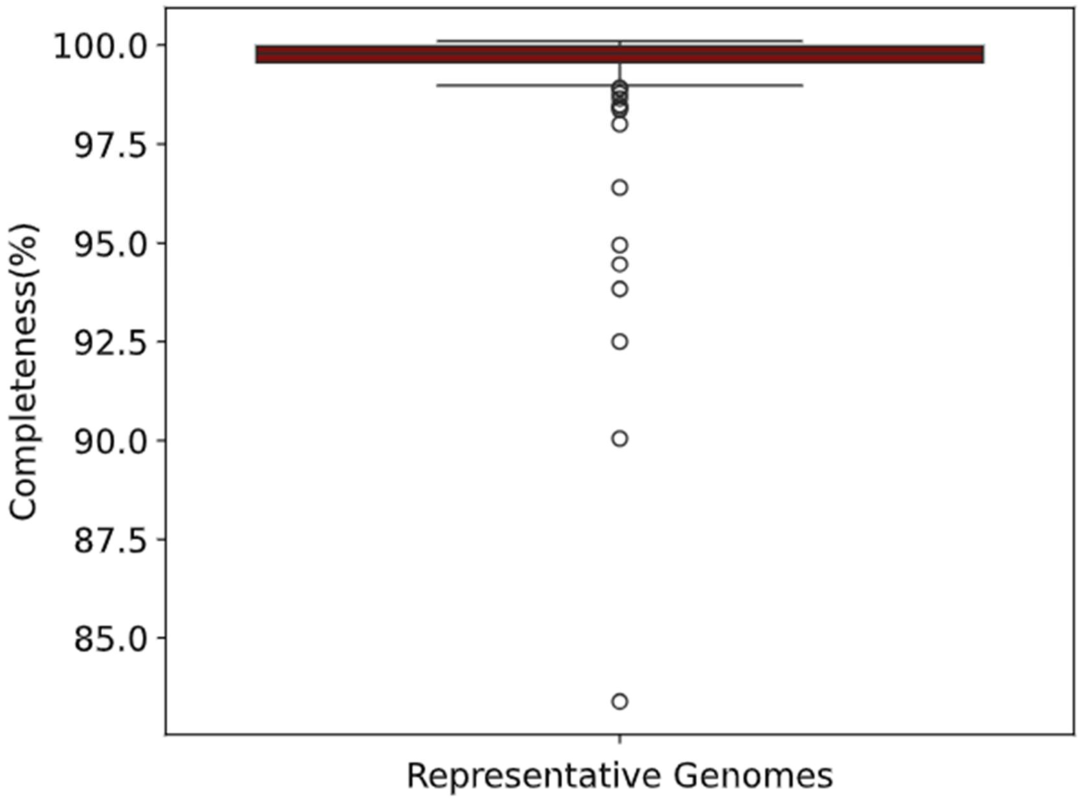

Figure S3 . Assembly of *in silico* long reads converted to short reads. In silico long reads were generated for a set of 197 diverse phage genomes using PBSIM [19], converted into short 300 bp reads and assembled with SPades. Genome completeness was determined by comparison to the original genome sequence length.

Classic Phage Isolation & Sequencing

Plaque assay
[1-2 days] –Round 1

Plaque purification.
Rounds 2-3 [6 days] *#

Propagation of high titre stock
2-3 days * #

Phenol DNA extraction & Purification
2 days *

Commercial Sequencing
~ 14 days [minimum]
21-35 often the norm
No hands-on time

Genome Assembly 1 day
Identify if single phage or multiple phages present

1 phage plaque:
26+ days minimum to obtain a single genome sequence

Time estimates are based on using *E.coli* , for comparison with the *Plaque-2-seq* method where 96 plaques were sequenced in 5 days.

#Potential significant time savings are obtained using *Plaque-2-seq* for slower growing organisms where further time is required for plaques to develop and propagate the bacterial hosts. A phage genome can be rapidly sequence and evaluated to determine if further purification or the isolate is identical to another phage

*time required increases with increasing number of phages

Plaque-2-seq

Monday

Plaque assay
[1-2 days]

Tuesday

Pick plaques
DNase I treatment
Multiple displacement amplification
S1 nuclease step
Library preparation and load sequencer
[1 day]

Wednesday-Thursday

Sequencing - no hands on time

Friday

Assembly
[1 day]. Identify if single phage or multiple phages are present

1 phage plaque:
5 days to obtain single genome sequence
96 plaques : 5 days to obtain phage genome sequences. Validated with *E. coli* phages in this study

Figure S4 Comparison of classic phage isolation and sequencing to *Plaque-2-seq* timelines

Plaque-2-seq optimisation overview

Conditions used for sequencing 96 plaques

DNA amplification

Conditions tested :
1, 0.5 , 0.3, 0.25 volumes of manufactures recommended volumes. No significant difference between conditions on DNA yield

Recommendation:
If cost reduction is a priority – use minimal volume of EquiPhi.

→ 0.25 volumes of manufactures recommend EquiPhi polymerase

DNase I treatment

Conditions tested :
With and without treatment of samples with DNase I. No detectable significant difference in host reads was detected.

Recommendation:
Use DNase I , as it didn't have adverse effects. Process works if excluded.

→ DNase I treatment

S1 nuclease treatment

Conditions tested :
With and without treatment of samples with S1 nuclease. S1 nuclease significantly reduced chimeric reads.

Recommendation:
Use S1 nuclease. Process works if excluded. Higher proportion of reads need chimera detection if excluded

→ S1 nuclease treatment

Read correction and assembly

Conditions tested : Flye assembly with long reads v Flye assembly with chimera correction of long reads (SACRA).
Spitting of long reads into short reads and SPAdes assembly.

Recommendation:
Create short reads and utilise SPAdes. Significant time savings when using 96 barcodes.
DO NOT use long reads without chimera correction.

→ Create 300 nt short reads from long reads and assembled with SPAdes

Figure S5 Optimised Workflow

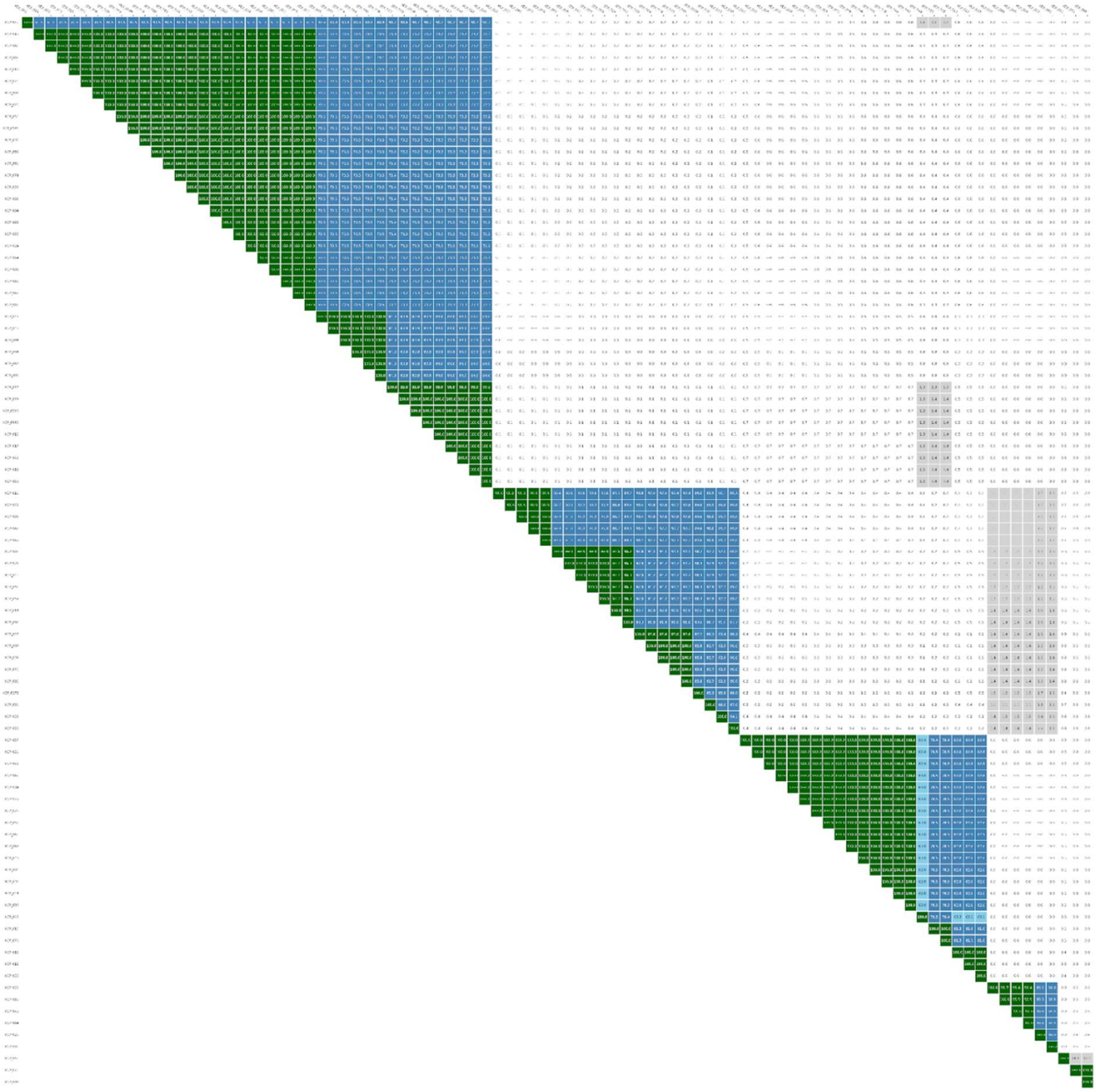

Figure S6. Comparative Genomics of Coliphages. All coliphages were compared using *taxMyPhage* using the similarity option.
